# Surface Texture-Based Surface Treatments on Ti6Al4V Titanium Alloys for Tribological and Biological Applications: A Mini Review

**DOI:** 10.3390/ma11040487

**Published:** 2018-03-24

**Authors:** Naiming Lin, Dali Li, Jiaojuan Zou, Ruizhen Xie, Zhihua Wang, Bin Tang

**Affiliations:** 1 Research Institute of Surface Engineering, Taiyuan University of Technology, Taiyuan 030024, Shanxi, China; lidali0197@link.tyut.edu.cn (D.L.); zoujiaojuan@tyut.edu.cn (J.Z.); tangbin@tyut.edu.cn (B.T.); 2 Shanxi Key Laboratory of Material Strength and Structure Impact, Taiyuan University of Technology, Taiyuan 030024, Shanxi, China; wangzhihua@tyut.edu.cn; 3 Department of Chemical and Materials Engineering, University of Alberta, Edmonton, AB T6G 1H9, Canada; 4 Department of Civil Engineering, Taiyuan University of Technology, Taiyuan, 030024, Shanxi, China

**Keywords:** surface texture, surface treatment, Ti6Al4V alloy, tribology, biology

## Abstract

Surface texture (ST) has been confirmed as an effective and economical surface treatment technique that can be applied to a great range of materials and presents growing interests in various engineering fields. Ti6Al4V which is the most frequently and successfully used titanium alloy has long been restricted in tribological-related operations due to the shortcomings of low surface hardness, high friction coefficient, and poor abrasive wear resistance. Ti6Al4V has benefited from surface texture-based surface treatments over the last decade. This review begins with a brief introduction, analysis approaches, and processing methods of surface texture. The specific applications of the surface texture-based surface treatments for improving surface performance of Ti6Al4V are thoroughly reviewed from the point of view of tribology and biology.

## 1. Introduction

Titanium (Ti) was once considered a rare metal, however, Ti ranks as the ninth Clarke number, it is the fourth most abundant structural metal on the earth, and Ti presents a level of about 0.6% in the world [1]. Titanium and titanium alloys have been rapidly developed since the pure metal first became commercially available about sixty years ago [1]. Thanks to their extraordinary merits of high strength to weight ratio, relatively low modulus, high yield strength and toughness, excellent corrosion resistance as well as promising biocompatibility, titanium alloys have received extensive attention in various fields ranging from civilian products to military equipment for decades [2,3,4,5]. Series of titanium alloys have been designed and produced for different purposes with great success. Ti6Al4V alloy (referred to Ti6Al4V hereafter) which was developed and made its name in the 1950s, was the first practical titanium alloy in the world. Ti6Al4V got its reputation of ace titanium alloy from the fact that most other existing titanium alloys for various applications were obtained by optimizing and improvement based on it. Up to now, Ti6Al4V is still the most frequently and successfully used titanium alloy, it occupies about one half of the total world production of titanium alloys [6,7,8]. However, Ti6Al4V cannot meet all of the engineering demands, e.g., it is seldom operated in tribological-related engineering conditions due to its drawbacks of low surface hardness, high coefficient of friction, and poor abrasive wear resistance. These shortcomings have greatly limited or even prevented Ti6Al4V larger scale use for various applications [9,10,11,12,13]. It is well known that degradation/failure of materials in engineering, e.g., wear and/or corrosion are mainly determined by the surface performance of the material rather than by bulk properties. Therefore, endowing improved properties on the surfaces of materials by surface modification technologies are attractive and suitable approaches to overcome the aforementioned issues [14]. Appropriate surface treatment is able to improve the surface performances (hardness, chemical stability, friction-reduction or wear-resistant) and retain the desirable bulk attributes of the materials, and then further expand the applications of materials in different fields [15]. On the other hand, the surface modification can also make a promising compromise between the cost and the performance of engineering components [16]. A variety of surface modification technologies have been applied to enhance the tribological performance of Ti6Al4V on the surface by forming coatings/films/layers [17,18,19,20,21,22,23,24,25,26,27,28,29,30,31,32,33,34].

**Figure 1:**
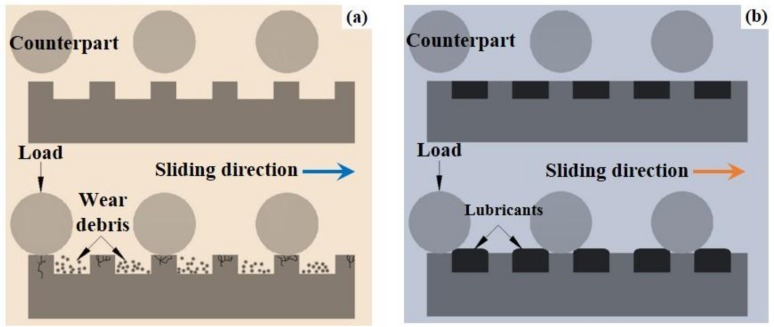
Schematic diagram of active roles of surface texturing in tribological performance: (**a**) continuous lubrication and (**b**) capturing friction debris.

According to recent bionic studies, apart from conventional surface modification technologies, appropriate design on the surface topography/pattern is able to improve the tribological performance of materials [35]. From the earlier point of view, smoother surfaces should be more favorable to improve the tribological performance of engineering components. However, recent achievements of bionics have suggested that non-smooth surfaces with regular arranged topographies/patterns usually exhibit promising tribological behaviors. For examples, dung beetle with non-smooth epidermis is able to resist wear and extrusion; the non-smooth skin of dolphin can effectively reduce its swimming resistance; the micro-rhombus structures on the surface of shark skin contribute to noise reduction in the process of diving [36,37,38,39]. The non-smooth surfaces in the natural world have brought about many meaningful inspirations to material scientists and engineers [40,41,42]. It has been confirmed that artificial design on the surface topography/pattern of materials by imitating the non-smooth surfaces in the natural world can even realize some similar functions [35,36,37,38,39]. The way to obtain artificial surface patterns with typical distributing characteristics such as dimples, grooves, pimples and so on were collectively named as surface texture (ST) [43,44,45]. Surface texturing which has been confirmed as an effective method to improve the tribological behaviors of materials and tools in tribology-related fields, has been considered a hot issue in material science and mechanical engineering over the last decade [46,47,48,49,50,51,52,53,54,55,56]. In general, the active roles of ST in tribological performance lie in the main aspects as follows (see Figure 1) [57,58]: service as storage of the solid/grease lubricant to provide continuous lubrication and improve the elastohydrodynamic effect under liquid lubrication; trapping wear debris generated during service and minimizing abrasive wear, reduction in nominal contact area. ST has been extensively applied on various materials for different purposes [52,53,59,60,61,62].

**Figure 2:**
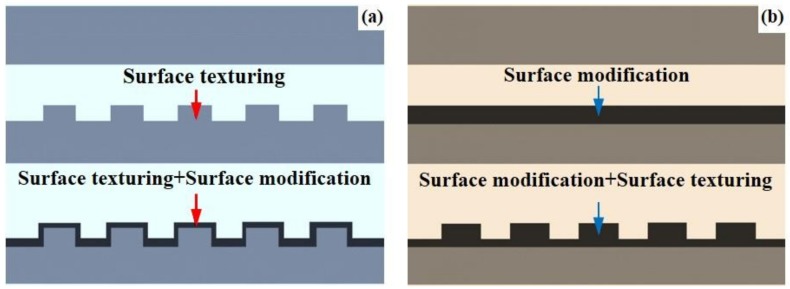
Schematic diagram of duplex treatment: (**a**) type one: surface texturing + surface modification; (**b**) type two: surface modification + surface texturing.

**Figure 3:**
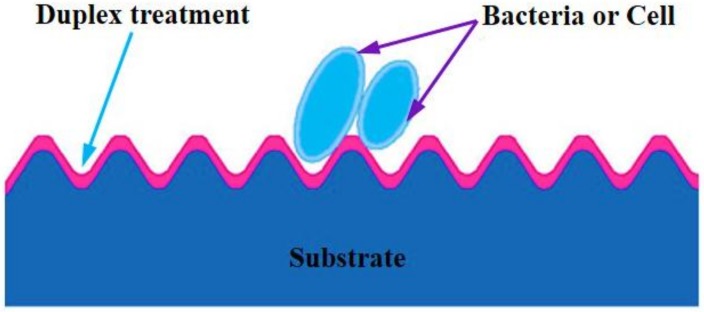
Schematic diagram of adhesion of bacteria or cell on a duplex treated surface.

With the advantages of surface modification technologies and surface texturing in improving the tribological performance of materials, some duplex treatments of surface modification-surface texturing have been developed. One kind is the “surface texturing + surface modification”, the other kind is the “surface modification + surface texturing”, as shown in Figure 2 [63,64,65,66,67,68,69,70,71,72,73,74,75,76]. These works have created a database and provided reference information for practical applications of surface modification-surface texturing duplex treatments. Due to its promising merits above, Ti6Al4V has long been used as hard tissue replacements (e.g., dental implant and artificial joint) and services in the human body. Before an implanting operation, Ti6Al4V biomedical devices and components are usually surface modified to achieve the expected properties of better biocompatibility, excellent antibacterial property, promising osteogenesis capability, low biotoxicity, benefiting cell adhesion and proliferation, controllable ion release rate, and so on [77,78]. The surface modification–surface texturing duplex treatment is considered to make use of the inherent positive effects of the surface modification layer as well as take the advantages of surface texture. Meanwhile a higher specific surface area which is favorable to hydrophilicity or hydrophobicity, adhesion and proliferation of cell and antibacterial behavior, might be realized on the surfaces of Ti6Al4V biomedical devices and components after the mentioned duplex treatment (Figure 3) [79]. It seems that the combination of the surface modification and surface texturing can achieve a “1 + 1 > 2” effect on improving the surface performance of Ti6Al4V.

Surfaces with promising super-hydrophobicity have received much attention from scientists due to their great potential in many applications, particularly in fields such as waterproofing, antifouling, self-cleaning, anti-corrosion, drag-reducing, anti-frosting, and anti-icing [80,81]. As a multi-purpose material, Ti6Al4V has also been endowed with super-hydrophobicity by surface texturing in some cases of the mentioned applications [80,81].

The relevant information with emphasis on the latest progress in the research on the surface texture-based surface treatments on Ti6Al4V for tribological and biological applications is summarized in this mini review. Although the surface texture-based surface treatments are not fire-new technologies, the demand for failure protection and to prolong service life drive the on-going interests of the scientific community making them still worthy of further studies. This work is expected to create a database and provide reference information, thereby broadening the practical applications of surface texture-based surface treatments on Ti6Al4V and other metallic materials.

## 2. Surface Texture-Based Surface Treatments with Improved Performance

Based on the above discussed background, it has been confirmed that ST possesses outstanding advantages. Therefore, many researchers have dedicated themselves to the studies of surface texture, including the characterizations, fabrications, and applications of surface texture. Surface patterns on textured surfaces usually have regular distributing characteristics at nano- or micro-scales, therefore microscopic analysis is required. Scanning electron microscope (SEM), atomic force microscope (AFM) and laser scanning confocal microscope (LSCM) are often used to observe the geometrical characteristics of surface texture [82,83,84]. Additionally, some researchers have paid attention to the residual stresses in surface layers on the textured surfaces [85,86].

With respect to the research on fabrications of surface texture, researchers mainly focus on the shapes of the ST unit, the processing methods of surface texturing, and related parameters [87,88,89,90,91,92,93]. Dimple and pimple with geometric configurations of ellipse, circle, triangle, square, hexagons, and grooves in the straight and zigzag lines have been adopted to form the shape of ST [40,41,42,43,44,45,87]. Up to now, there have been several ways to obtain ST on the surfaces of materials [41,94], including laser surface texturing (LST), laser shock peening (LSP), electro spark surface texturing (ESST)/electrical discharge machining (EDM), chemical reactive ion etching (RIE), lithography and anisotropic etching (LIAE), abrasive jet machining (AJM), lithography, galvanoformung, and abformung (LIGA), vibrorolling, undulated surfaces and so on. Actually, amongst all the practical surface texturing methods, it seems that LST is the most promising concept. This is because the laser is extremely fast and allows short processing times, it is clean to the environment, and provides excellent control of the shape and size of the micropatterns, which allows realization of optimum designs [40,41,42,90]. Generally, the fabricating parameters of ST are case-by-case. Taking LST as an example, the width and depth of the textures are related to scanning velocity, power, frequency, and pulse width of the laser [93]. On the other hand, the distribution characteristic (area density: the ratio of the ST area to the whole surface area) of ST play an important role in the surface performance of a textured surface. Depending on the specific situation, parameter optimization of surface texturing is usually conducted through trial and error tests [87,88,89,90,91,92,93,94]. The existing applications in tribology and biology of surface texture-based surface treated Ti6Al4V were reviewed, and the relevant research on fabrications of ST are also presented.

In the following, surface texture-based surface treatments, including the single surface texturing and surface texturing/surface modification duplex treatment on Ti6Al4V alloy for tribological and biological applications are suggested in Sections 2.1 and 2.2.

### 2.1. Tribological Applications

Under different service conditions, a friction phenomenon usually exists between relative movement interfaces of solid–solid, solid–liquid and solid–gas [95,96,97]. Friction normally can lead to damage of wear and/or fatigue, lowering work efficiency and producing noise pollution when the engineering components are used under different service environments where the above-mentioned friction interfaces are involved [22,98,99]. While under the negative effects of friction in normal applications, the service performance of Ti6Al4V engineering components would be decreased and then the lifecycle of the whole equipment might also be reduced [100,101]. Surface texture-based surface treatments developed by different scientists and engineers have provided promising approaches to overcome the relevant issues [42,43,44,45,46,47,48,49,50,51,52,53,54,55,56,57,58,59,60,61,62,63,64,65,66,67,68,69,70,71,72,73,74,75,76,77,78,79,80,81,82,83,84,85,86,87,88,89,90,91,92,93,94,102,103].

Guo and Caslaru [90,101] conducted a novel micro laser shock peening (LSP) on Ti6Al4V to fabricate micro-circle dent arrays with different densities based on a surface patterning technique at various power levels, 1 W, 2 W, and 3 W. It was found that higher laser power could produce deeper dent. The highest depth of approximately 1 µm was achieved using a 3 W laser power, and the dent diameter also increased as the laser power was increased. A pile-up region appeared on the outer edge of each dent. It was believed that tensile stress developed in the pile-up region. Meanwhile the LST treatment could create a hard surface, the hardness in the central zone of the dent was enhanced by about 15%. Strain hardening, strain-rate hardening, and compressive residual stress (an increase in compressive residual stress on the LSP treated surface was found after removing the pile-up effect via mild polishing) contributed to the hardening effect in the dented area. Two surfaces with dent densities of 10% and 20% were selected to investigate the tribological behaviors under flooded and boundary lubricated conditions, countered with chrome steel balls. The 20% dent density produced a higher pile-up zone density which indicated a negative influence on tribological behavior. However, the beneficial effects of surfaces with 10% dent density were more pronounced at reducing the coefficient of friction (CoF) and wear rate under both lubricated conditions. The results confirmed that LSP is a reliable process for fabricating micro-dent arrays with different densities, micro-dents with low density were suitable for Ti6Al4V in sliding contact applications.

In order to improve the tribological performance of Ti6Al4V, Hu et al. [104] applied laser surface texturing (LST) to form regular circle dimple ST with three different diameters of 45 μm, 160 μm and 300 μm, as well as with a spacing of 100 μm and a depth of 25 μm on Ti6Al4V. Two types of poly-alpha-olefin (PAO) lubricants with different kinetic viscosities were evaluated as lubricants on raw Ti6Al4V and LST treated Ti6Al4V samples using a pin-disc tribometer and steel pins. It was observed that the textured surfaces exhibited lower friction coefficients and wear compared with the un-textured surface. Furthermore, the textured surfaces exhibited better tribological properties when the tests were conducted under higher speeds and loads and with higher viscosity oil. However, when the load increased, the effect of friction reduction for all the textured surfaces decreased for the lubricating oil with lower viscosity. It was found that the lubricant film thickened due to the enhancement of hydrodynamic pressure near the dimples, and higher viscosity oil was prone to provide a secondary lubrication effect. The formed micro-dimples on Ti6Al4V surfaces shifted the transition from boundary to mixed lubrication as the tests were operated under much higher loads when higher viscosity oil was used. The effect of dimple sizes on the tribological properties the LST surfaces under the same testing conditions was also analyzed. The results showed that the dimple with a diameter size of 160 μm was more favorable to reduce friction when the interval of dimples was fixed.

By using LST technique, Lian et al. [105] fabricated three kinds of ST on Ti6Al4V surfaces: groove, crosshatch (included angle 90°), and dimple with interval spacing of 100, 200, and 300 μm, respectively. All of the LST treated surfaces showed higher surface hardness values than that of the raw Ti6Al4V. Actually, the hardness values could be arranged as follows: raw Ti6Al4V < groove-Ti6Al4V < crosshatch-Ti6Al4V < dimple-Ti6Al4V. It was seen that the dimple textured samples with interval spacing of 200 and 300 μm had remarkably reduced the coefficient of friction, while groove and crosshatch textures were helpful for friction reduction. The textured surfaces presented shallower wear traces than that of the raw Ti6Al4V, the distribution characteristics of the three kinds of ST received much slighter damage compared with the untreated Ti6Al4V. In this work, a conclusion could be drawn that groove, crosshatch (included angle 90°), and dimple obtained by LST managed to improve the wear resistance of Ti6Al4V.

Xu et al. [106] manufactured crosshatch STs on Ti6Al4V surfaces by employment of electrical discharge machining (EDM). The orthogonal design was applied to investigate the effects of geometric parameters (width, depth, interval/width ratio, and angles) of the crosshatches on the tribological characteristics of Ti6Al4V alloy in water lubrication against Si_3_N_4_. The width, depth, interval/width ratio, and angles of the crosshatch were factored in, and each of the above factors had four levels: width values (0.15, 0.20, 0.25, 0.30 mm), depth values (0.05, 0.075, 0.1, 0.125 mm), interval/width ratio (2, 6, 10, 14) and angles (15°, 30°, 45°, 60°). The results suggested that the crosshatch ST with suitable geometric parameters could effectively reduce the friction coefficient and wear rate of tribopairs in water lubrication. It was found that width (0.2 mm), depth (0.125 mm), interval/width ratio (10) and angle (45°) were the optimum factors and levels for realizing a lower and stable friction. While the combinations of factors and levels with width (0.2 mm), depth (0.05 mm), interval/width ratio (2), and angle (45°) revealed the lowest wear loss. When the angle of the crosshatch was 45°, both the friction coefficient and the wear of the tribopairs simultaneously decreased. The extent of the geometric parameters impact on friction coefficient was arranged in the following sequence: width > depth > angles > interval/width ratio. The depth and angle of the crosshatch were the main factors which influenced the wear loss of the tribopairs.

Bonse et al. [107] obtained homogeneous ripple ST with a spatial period of about 600 nm on Ti6Al4V surface after multiple femtosecond laser pulse irradiation. Compared to the blank sample, the ripple textured Ti6Al4V demonstrated lower and more stable friction coefficient in reciprocal sliding against a hardened steel ball under engine oil lubrication. The treated Ti6Al4V received a shallow and narrow wear trace, which was also hardly visible by microscope. Slight surface damage was generated after the tribological test. Meanwhile, it was found that the additives in the engine oil efficiently covered the ST, where a complete gliding intermediate layer was formed. This intermediate layer which played an important role in reducing friction and wear during the reciprocal sliding motion, prevented direct intermetallic contact of the metallic tribopairs.

Lian et al. [108] concentrated on enhancing the tribological performance of Ti6Al4V in seawater and built crosshatch and dimple textures on Ti6Al4V surfaces via LST. Tribological performance was evaluated by reciprocating friction tests against Si_3_N_4_ balls in artificial seawater and distilled water, respectively. The results showed that the friction coefficients and wear losses in volume of crosshatch and dimple textured Ti6Al4V surfaces were far smaller than those of the raw Ti6Al4V substrates. The friction coefficients belonging to the surfaces with crosshatch and dimple were decreased by 11.7% and 17.8%, and wear losses in volume were reduced by 57.5% and 36.8% in artificial seawater. Both textured Ti6Al4V surfaces revealed lower friction coefficients in artificial seawater than those in distilled water, while the wear losses in volume led to the opposite results. Ti6Al4V was prone to form a passive film on the surface when it was put in artificial seawater, the passive film could act as a solid lubricant due to its lower shear strength. Meanwhile artificial seawater was more benefit for the passivation process than distilled water, therefore all the Ti6Al4V samples showed lower friction coefficients in artificial seawater compared with the samples which were measured in distilled water. However, passivation–depassivation which continuously took place on the surfaces of Ti6Al4V during the reciprocating friction tests, could significantly lead to material removal on the surfaces. That was why the Ti6Al4V samples presented lower friction coefficients but higher wear losses in volume in artificial seawater than those in distilled water. Fortunately, the tribological performances of Ti6Al4V in artificial seawater were dramatically improved as the crosshatch and dimple STs were prepared, as expected.

Further, Lian et al. [109] first fabricated crosshatch and dimple ST (with a spacing of 100 μm) on Ti6Al4V by LST, and then both of the textured surfaces were coated with nano SiO_2_ particles via the sol-gel method. The ultimately obtained surfaces (SiO_2_-crosshatch and SiO_2_-dimple) indicated excellent super-hydrophobic property. The super-hydrophobic property of SiO_2_-crosshatch textured surface was a bit better than the SiO_2_-dimple texture surface. Meanwhile the subsequent measurements of tribological performance under dry sliding demonstrated that the wear rates of the SiO_2_-crosshatch textured surface and SiO_2_-dimple textured surface were decreased by 53.8% and 32.3%, respectively. While both of the fluctuation and friction coefficient values decreased on SiO_2_-crosshatch textured and SiO_2_-dimple textured surfaces. Ti6Al4V samples were well endowed with promising tribological property on surfaces using the adopted duplex treatments.

By employment of the LST method, Hu et al. [110] obtained three dimple STs on Ti6Al4V. The geometric parameters were: diameter ~150 μm, average depth ~40 μm and three dimple intervals were designed by controlling the dimple area densities (13%, 23%, and 44%). Meanwhile the formed STs were burnished using commercially available MoS_2_ solid lubricant. Under dry sliding against bearing steel, it was found that the textured Ti6Al4V with higher dimple density showed lower friction and wear compared with blank Ti6Al4V only if the tribological tests were conducted under low load and speed. ST with higher dimple density led to a lower friction coefficient and also contributed to a more promising wear debris capturing effect. As the LST surfaces were burnished MoS_2_ film, all of them exhibited excellent friction-reducing and wear resistance under any applied load. It was considered that by transferring of MoS_2_ reserved in dimples, into the space between the dimples, a continuous solid lubricant film was maintained on the surface. The textured surfaces with 23% dimple density revealed the lowest friction coefficients under different testing conditions. However, a longer wear lifetime was realized by increasing the dimple density, which can be ascribed to the higher remaining amount of MoS_2_ and higher transferring efficiency of MoS_2_ from the dimples to the friction interface.

Ripoll et al. [67] used a Nd:YAG nanosecond pulsed laser to form hexagonally arranged dimples STs (with diameter of 40 μm, depth of 16–20 μm, spacing of 40, 50, 60, and 70 μm) on Ti6Al4V. The blank Ti6Al4V and textured Ti6Al4V were coated with MoS_2_ films with a thickness of 2 μm by sputtering. Dry reciprocating sliding tests were performed on MoS_2_-coated textured Ti6Al4V and MoS_2_-coated Ti6Al4V using a ball on flat configuration (against 100Cr6 steel balls) at two different oscillation amplitudes. The results displayed that under certain conditions, surface texturing was able to reduce friction, prolonged the lifetime of the film, and gave progressive film degradation until failure. It was seen that the friction coefficients significantly decreased, especially for higher dimple densities owing to the better wear debris capturing function. With respect to low amplitudes, ST could effectively act as wear debris traps and then increase the lifetime of MoS_2_ film. However, in respect of high amplitudes, ST had a negative impact on tribological behaviors. Additionally, aiming to avoid yielding excessive contact pressure and low amount of MoS_2_, the normal distance between dimples was preferred to be not smaller than 50 μm. For the testing amplitude of 0.5 mm, an optimum dimple density was found to be between 40% and 67%.

Auezhan Amanov [111] produced dimple ST with diameter (100 μm) and depth (25 μm) on Ti6Al4V by LST, and then a Cr-doped diamond-like carbon (DLC) film was successively fabricated on the dimple textured Ti6Al4V via unbalanced magnetron sputtering (UBMS). The tribological characteristics of the un-textured (polished), dimple textured and coated dimple textured Ti6Al4V samples were investigated against Cr-plated bear steel SAE 52100 pin at 50 ℃ under poly-alpha-olefin (PAO) oil lubricating conditions. It was seen that a noticeable enhancement in hardness and H/E ratio of the Ti6Al4V after LST process was found, which meant the dimple textured Ti6Al4V had higher mechanical properties and resistance to plastic deformation. This was ascribed to the microstructural modification due to the high energy action of the pulsating laser beam. At normal loads of 10 N and 20 N, the friction coefficient of the coated dimple textured Ti6Al4V was reduced by about 67% and 50%, and 75% and 65% in comparison to those of the un-textured and dimple textured samples, respectively. It was found that the coated dimple textured Ti6Al4V specimen showed significantly lower wear rate compared with those of the un-textured and dimple textured samples for the applied normal loads. The wear rate of the un-textured specimen was slightly higher than that of the dimple textured specimen and the coated dimple textured Ti6Al4V showed far lower wear rate than those of the un-textured and dimple textured samples. After LST + deposition of Cr-doped DLC film, Ti6Al4V benefited a lot from the functions of ST: storage of oil and wear debris capturing, as well as the merits of DLC: high hardness and H/E ratio.

Micro arc oxidation (MAO) has long been applied to improve the tribological performance of Ti6Al4V with great success by receiving series of ceramic coatings on the surface [15,24]. Wang et al. [70] employed a fine particle shot-peening (FPSP) process to form half-ball dimple ST on Ti6Al4V using ball-shape γ-Al_2_O_3_ particles with an average diameter of 28 μm to avoid introducing iron pollutants. The half-ball dimples distributed on the FPSP treated Ti6Al4V substrate surface resulted in an obvious increase in surface roughness from Ra 0.26 μm to Ra 1.65 μm. The pretreated process of FPSP was followed by micro arc oxidation (MAO) treatment. The related samples in that work were original Ti6Al4V (polished), FPSP-Ti6Al4V, MAO coatings (with thickness of 5 μm and 10 μm: MAO5 and MAO10) and FPSP-MAO coatings (with thickness of 5 μm and 10 μm: FPSP-MAO5 and FPSP-MAO10). The tribological behaviors of the mentioned samples were investigated on a pin-on-disk tester against SAE 52 100 steel ball (with a diameter of 6.0 mm, a surface roughness Ra 0.05 μm and a hardness HRC 61) with normal load of 1 N under dry sliding condition. There was no obvious difference in variation trends of friction coefficient between original Ti6Al4V FPSP-Ti6Al4V. However, both of the MAO coatings and FPSP-MAO coatings showed no obvious friction reduction effect on Ti6Al4V, which was in good agreement with the previous publications [32,112]. The MAO5 and MAO10 coatings were completely worn out after 900 and 1700 sliding cycles implying equal friction coefficient values of MAO coatings and original Ti6Al4V. Throughout the sliding cycle number, there was no evidence that could reveal complete worn damage of FPSP-MAO5 and FPSP-MAO10 coatings. The dimples on the surfaces of FPSP-MAO5 and FPSP-MAO10 coatings could act as sink for trapping wear debris, which was helpful to alleviate three body wear, while the restrained three body wear was responsible for the enhancement in wear resistance of FPSP-MAO5 and FPSP-MAO10. The resultant specific wear volume of each tested sample could be arranged as follows: original Ti6Al4V > FPSP–Ti6Al4V > MAO5 > MAO10 > FPSP–MAO5 > FPSP–MAO10. FPSP–MAO was considered as an effective means to improve the wear resistance of Ti6Al4V. Meanwhile the fatigue life of FPSP-Ti6Al4V sample was increased by 39% in comparison to the original Ti6Al4V, which was ascribed to the compressive residual stress caused by FPSP treatment. With respect to the simples of MAO5 and MAO10, the fatigue life decreased from 20, 618 cycles for MAO5, to 16, 282 cycles for MAO10. While in respect of the FPSP-MAO samples, the fatigue life decreased from 23, 223 cycles for FPSP-MAO5, to 17, 653 cycles for FPSP-MAO10. At the same thickness of 5 μm and 10 μm, FPSP-MAO5 enhanced fatigue life by 12.6% compared with simple MAO5, while FPSP-MAO10 suggested a slight improved fatigue life only by 8.4% compared with sample MAO10. In general, the FPSP-MAO coatings presented better fatigue resistance in cyclic loading conditions. Nevertheless, because of the fragile nature of the ceramic coatings, all the MAO coated samples exhibited deterioration in fatigue lives.

Using the LST technique, Prem Ananth et al. [113] attained dimple ST with geometric parameters of: dimple densities of 38%–42%, diameter of 100 μm, depth of 2.5 μm, and the interval between two consecutive dimples was 160 μm. Magnetron sputtered physical vapor deposition (MSPVD) technique was applied to prepare nano AlCrN composite coatings on raw and dimple textured Ti6Al4V surfaces. Due to the positive mechanical locking effect, the coating cover on the LST Ti6Al4V surface showed appreciable improved bonding and shear strength. Under different normal loads (9.8–29.7 N), the LST + MSPVD treated Ti6Al4V suggested more stable friction coefficients and longer wear lives than that of the AlCrN coating on raw Ti6Al4V. Up to now, LST has been usually followed by advanced surface modification technologies to improve the surface performance of Ti6Al4V. Prem Ananth et al. [114] used to produce dimple ST on Ti6Al4V surface with geometric parameters of: dimple density (38%–42%), diameter (100 μm), depth (2.5 μm), and dimple-dimple interval (160 μm). The blank and dimple textured Ti6Al4V samples were coated with titanium aluminum nitride (TiAlN) nanocomposite coatings with chromium interlayer via cathodic arc physical vapor deposition (CAPVD) system. The obtained coatings reached a total thickness of 4–5 μm. Using scratch tester and pin-on-disc tribometer, the original Ti6Al4V, dimple textured Ti6Al4V and TiAlN coated samples were subjected to bonding strength and tribological evaluations. It was found that the bonding strength of the coating on dimple textured Ti6Al4V with a chromium inter layer increased by 8% compared to that of the coating on the original Ti6Al4V surface. Under dry sliding contact conditions (variation in load: 9.8 N, 14.7 N, 19.6 N, and 29.4 N), increasing normal load led to an increasing friction coefficient, however the TiAlN coated dimple textured Ti6Al4V presented slightly lower increasing rates in comparison to the TiAlN coating on original Ti6Al4V. Meanwhile it was seen that samples with textured surface revealed comparatively lower steady friction coefficients compared to those of lapped surfaces. It was found that increasing normal load contributed to early coating failure and then resulted in metal-to-metal contact. However, it was confirmed that the LST + CAPVD treatment could significantly improve the tribological performance of Ti6Al4V alloy.

Qin et al. [115] investigated the tribological properties of LST treated and plasma electrolytic oxidation (PEO) duplex-treated Ti6Al4V deposited with MoS_2_ film. First, the dimple ST formed a Ti6Al4V plate surface using a pulsed Nd:YAG laser with a wave length of 1064 nm and a pulse width of 450 ns. Then the LST-Ti6Al4V plate was ground by 800 mesh SiC emery paper to remove the raised ripples. The electrolyte then used for PEO contained 12 g/L sodium aluminate (NaAlO_2_), 1.6 g/L trisodium phosphate (Na_3_PO_4_·12H_2_O) and a small amount of sodium hydroxide (NaOH). The PEO treatment was conducted for 1 h with an initial voltage of 440 V, adopting a duty cycle of 20% and a frequency of 400 Hz to form PEO-Ti6Al4V and LST + PEO-Ti6Al4V. The temperature of electrolyte was maintained at about 30 ℃ through a water cooling system. The lubricating MoS_2_ film was bonded onto the tested samples using E44 epoxy and acetone as the adhesion agent and the solvent, and the acquired samples were MoS_2_-Ti6Al4V, LST + MoS_2_-Ti6Al4V PEO + MoS_2_-Ti6Al4V and LST + PEO + MoS_2_-Ti6Al4V. It was seen that micro dimples with a diameter (D) of 260 μm, an interval between the centers of two dimples of 500 μm, and an area density (s) of 21.2% were regularly distributed on the polished Ti6Al4V surface. The PEO coatings on both the un-textured and LST-Ti6Al4V surfaces reached a similar thickness of 30 ± 5 μm.

Friction and wear tests were conducted using a ball-on-disk tribometer under dry sliding conditions at the applied loads of 0.3–5 N and a constant velocity of 0.1 m/s against a GCr15 steel ball with a diameter of 4 mm. Under a load of 5 N, polished Ti6Al4V substrate suffered severe adhesive wear and plough, the dimples on the LST-Ti6Al4V were nearly worn out and some wear debris was trapped in the residual dimples. The predominant wearing modes of PEO-Ti6Al4V were deformation and abrading. LST + PEO - Ti6Al4V received the lightest damage, its worn trace, a mild polishing of the raised rims of dimples, occurred on the space between the micro-dimples in the sliding direction. The wear rate of polished Ti6Al4V (4.27 × 10^−4^ mm^3^/Nm) was slightly above the LST-Ti6Al4V (3.93 × 10^−4^ mm^3^/Nm). The PEO-Ti6Al4V showed a lower wear rate of 1.88 × 10^−4^ mm^3^/Nm. It was observed that the LST + PEO - Ti6Al4V provided the most promising improvement in wear resistance of Ti6Al4V, indicated by the lowest wear rate of 8.45 × 10^−5^ mm^3^/Nm as compared with other samples. Meanwhile all of the samples benefited from the bonded MoS_2_ film in tribological performance varying in low friction duration. It just took 5 min to completely remove the surface of MoS_2_-Ti6Al4V under a load of 1 N. The LST + MoS_2_-Ti6Al4V survived for a longer period of 20 min with a lower friction coefficient than that of MoS_2_-Ti6Al4V under the same load. However even as the load was raised up to 5 N, the PEO + MoS_2_-Ti6Al4V still revealed a stable lower friction coefficient than MoS_2_-Ti6Al4V and LST + MoS_2_-Ti6Al4V for about 200 min. The LST + PEO + MoS_2_-Ti6Al4V presented surpassed friction behavior in comparison to PEO + MoS_2_-Ti6Al4V, as expected. The MoS_2_ which was stored in the dimple-reservoirs was squeezed out to the contact region and formed a thin lubricating transfer film on the friction interface during dry sliding. The thin lubricating transfer film was beneficial to friction reduction and was helpful to sustain longer duration. Additionally, the partially open micro-dimples could act as sinks for trapping wear debris, which was also beneficial in reducing the damage and prolonging the effective life of the lubricating film.

Furthermore, the effects of textured dimple area densities (referred to S, S = dimple area/total surface area) and the surface roughness values of oxide ceramic underlay which influenced the lifetime of MoS_2_ films were thoroughly studied by Qin et al. [116]. Similar results showed that the LST + PEO + MoS_2_-Ti6Al4V which was superior to the MoS_2_-Ti6Al4V, LST + MoS_2_-Ti6Al4V and PEO + MoS_2_-Ti6Al4V, exhibited much longer low friction life. It was obvious that the low friction life of the LST + PEO + MoS_2_-Ti6Al4V was prolonged with increasing the S values from 8% to 55%, due to a higher area density which could preserve a larger amount of MoS_2_ in the dimples. The lubricants were continuously and effectively replenished at a shorter interval between the dimples by adequate supply of fresh MoS_2_ both from the large textured dimples and the small discharged dimples during sliding and resulted in a longer sliding life with low friction. In addition, the hard and polished LST-PEO surfaces could also provide a high load support for the soft MoS_2_ film. The received LST + PEO + MoS_2_-Ti6Al4V (with S = 55%) with a Ra = 2.9 μm showed disappointing friction behavior. However, when the surface roughness values of LST + PEO + MoS_2_-Ti6Al4V were polished down to Ra = 1.6 μm and Ra = 1.0 μm, the obtained sample revealed the best friction behaviors. In this work, a much longer low friction life LST + PEO + MoS_2_-Ti6Al4V was realized by choosing an LST surface with S = 55% and polishing the LST + PEO + MoS_2_ to a surface roughness of Ra = 1.0 μm.

In He et al.’s work [64], STs of micro-dimples with various textured dimple area densities and diamond-like carbon (DLC) films were fabricated on the surfaces of Ti6Al4V by LST and close-field magnetron sputtering (CFMS), respectively. To be specific, the LST was conducted using a Nd:YAG laser with a wave length of 1064 nm, a frequency of 10 kHz and a 90% overlapping rate of laser spot. An average power of 10 W at a 5 mm/s traverse speed was applied to treat the samples. After LST, all the received samples (with textured dimple area densities of 13%, 24%, and 44%) were subjected to two step polishing processes, aimed to eliminate bulges or burrs around the edge of the dimples and obtaining a roughness of Ra ≤0.02 μm. High-energy ion implantation of nitrogen was performed on the textured and smooth Ti6Al4V samples to improve their surface hardness (pulse voltage −60 kV; frequency 60 Hz; pulse width 30 μs; working gas pressure 3 × 10^−2^ Pa). Subsequently, thin chromium films (220 nm thick) were deposited on the as-nitrogen-implanted samples as interlayers to improve bonding strength, and DLC films with a total thickness of 3.3 μm were prepared by CFMS using high-purity graphite targets at a DC power supply of 2500 W. Each DLC deposition process began at an original pressure of about 3 × 10^−3^ Pa, and the deposition was performed at a substrate pulsed bias of −70 V, a frequency of 250 kHz, and a process pressure of 0.2 Pa under Ar flow. The obtained specimens were marked as DLC-smooth, DLC-T13%, DLC-T24%, and DLC-T44%. Tribological tests were performed on a ball-on-plate reciprocating mode with a reciprocatory displacement of 5 mm, a normal load of 5N, a sliding frequency of 5 Hz, and the sliding direction parallel to the LST patterns. The Ø10 mm AISI 52100 steel balls, with hardness of HV 725, were used as counterparts. Dry friction and liquid lubrication (1-butyl-3-methylimidazolium hexafluorophosphate ionic liquid) conditions were conducted for 50,000 cycles with the same test parameters in ambient air (temperature of 20 ℃ and relative humidity of 35%).

The effects of dimple area densities and DLC phase transformation on the properties of Ti6Al4V under dry friction and liquid lubrication conditions were thoroughly investigated. The DLC-smooth suggested a critical load of 55 N, while the DLC-T13%, DLC-T24%, and DLC-T44% samples indicated almost the same critical load of about 37 N. Damage to the DLC films, particularly to the DLC-T44%, was more severe in comparison to DLC-T13% and DLC-T24% samples. The stress concentration in the films around the rims of the dimples resulted in low load carrying capacities. Due to the combined action of dimple-induced graphitizing transformation and the function of the fluid/wear debris reservoirs of the dimples, DLC-T44% always revealed remarkably lower friction coefficients than those of DLC-smooth and DLC-textured samples with lower dimple area densities regardless of under dry friction or under liquid lubrication conditions. However, the most outstanding wear resistance (with the lowest wear rate) under dry friction was observed on DLC-T24%, which could be explained in that an appropriate number of dimples on the DLC surface were not only trapped the most wear debris but also maintained film hardness (a low level of graphitization transformation) during friction. It was concluded that combining appropriate surface texturing with DLC films was able to effectively reduce the friction and wear of Ti6Al4V and thus could be beneficial for its wider applications.

Thermal oxidation (TO) is an ideal surface technology which has been extensively used to strengthen Ti-based materials. Its popularity is mainly assigned to its cost effectiveness, simplicity, and rapidity. Furthermore, the TO treatment has no special requirements for substrate geometric shape [117]. Taking the advantages of LST and TO processes, Sun et al. [118] formed a series of TO films of various thicknesses and composition on regular dimple textured Ti6Al4V surfaces. The STs on Ti6Al4V were realized by LST. The TO processes were performed at 500 ℃, 650 ℃, and 800 ℃ for 5–50 h. The LST treated Ti6Al4V were well covered with uniform and continuous TO coatings. It was also found that ST could decrease the internal stress and effectively improve bonding strength of the oxide film to the Ti6Al4V substrate. However, despite higher TO temperature and longer TO duration being favorable for increasing the thickness of the TO coatings, the obtained TO coatings under these conditions did not necessarily show excellent properties. Under dry friction conditions, TO-LST Ti6Al4V revealed much lower wear rates compared to the original Ti6Al4V under the same applied loads against GCr15 steel and two ZrO_2_ balls. The TO-LST Ti6Al4V surface received at 650 ℃ for 25 h suggested that the most excellent tribological properties were attributable to good comprehensive properties: a strongly bonded rutile coating with high hardness (7.73 GPa), increased hardness to elastic modulus ratio (0.096), and improved load-bearing capacity. The TO-LST Ti6Al4V obtained at optimal parameters with promising surface performance could withstand or weakened the wearing damage, on the other hand, the ST was able to capture the wear debris and then decreased the abrasive wear. The TO + LST duplex treatment indicated satisfactory complementation on improving tribological behavior of Ti6Al4V.

Martinez et al. [119] simultaneously realized thermochemical oxidation of UNS R56400 (Ti6Al4V) alloy through LST treatment in an open-air atmosphere with varied parameters. The treated UNS R56400 alloy indicated increased surface hardness and varied color tonality. The formation of titanium oxides and a rapid cooling rate contributed to the increase of hardness. The color tonality was especially affected by the laser treatment parameters of pulse rate (F (kHz)) and scan speed of the beam (Vs (mm/s)). Pin on disc tribological tests showed that design and development of topographies on the surfaces of UNS R56400 alloy were favorable for obtaining high sliding-friction-wear resistance. The most advantageous surface reduced the friction coefficient values by approximately 20%, which was obtained using a Vs of 150 mm/s.

As a special wearing mode, cavitation erosion which is fairly complicated, is usually generated under mechanical, chemical, and electrochemical interactions. Actually, cavitation erosion is a type of dynamic damage, and the fatigue damage on the material surface is caused by the impact of cavitation bubbles [120,121,122]. It has been confirmed that surface texture-based surface treatment certainly has positive effect on reducing cavitation erosion damage of Ti6Al4V. LST was conducted on Ti6Al4V by Pang et al. [123] to enhance its cavitation erosion resistance by forming groove, crosshatch (included angle 90°) and dimple STs with interval spacing of 50 μm and 100 μm. LST resulted in increasing surface hardness values of the textured Ti6Al4V samples by quenching effect (martensitic transformation and fine grain strengthening). The tested surfaces varied in surface hardness which could be arranged as: dimple (interval spacing of 100 μm) > crosshatch (interval spacing of 50 μm) > crosshatch (interval spacing of 100 μm) > groove (interval spacing of 50 μm) > groove (interval spacing of 100 μm) > original Ti6Al4V. By detecting the surface morphologies of the cavitation eroded samples, it was found that the dimple textured Ti6Al4V showed the best cavitation erosion resistance, followed by crosshatch textured Ti6Al4V, groove textured Ti6Al4V, and original Ti6Al4V. The arrangement in cavitation erosion resistance of the related Ti6Al4V samples was in good agreement with the results of surface hardness tests. Cavitation erosion resistance of Ti6Al4V specimens was significantly enhanced by LST.

On the basis of Pang et al.’ s work, Lian et al. [124] first fabricated groove and crosshatch (included angle 90°) STs on Ti6Al4V surfaces with interval spacing of 50, 100, 150 μm, and then prepared 1H, 1H, 2H, 2H-Perfluorooctyltrichlorosilane (FOTS) self-assembled monolayers (SAMs) on the blank and textured Ti6Al4V samples. The variations in surface hardness values of the textured Ti6Al4V were consistent with Pang et al.’s work: crosshatch textured Ti6Al4V showed higher surface hardness than that of groove textured Ti6Al4V. Furthermore, as the two textured surfaces were covered with FOTS-SAMs, both of them revealed better hydrophobicity than that of the original Ti6Al4V. By employment of microscopy observation, the original Ti6Al4V indicated a cavitation area of 75% on the total surface area. The crosshatch textured Ti6Al4V with different interval spacing revealed cavitation area values of about 18% (50 μm), 21% (100 μm), 25% (150 μm); the groove textured Ti6Al4V with different interval spacing presented cavitation area values of about 24% (50 μm), 29% (100 μm), 45% (150 μm). After FOTS-SAMs covering, both of the crosshatch textured and the groove textured Ti6Al4V with different interval spacing suggested far lower cavitation area, the largest one was only about 15%. FOTS-SAMs with hydrophobicity could reduce the flow resistance of water and then weakened the shock effect on the surface by bubble collapse. FOTS-SAMs on a textured surface were able to bring out more excellent cavitation erosion resistance of Ti6Al4V on the surface in comparison to a single FOTS-SAMs covering or single surface texturing treatment.

### 2.2. Biological Applications

Specially designed surface patterns (surface roughening) by surface texturing on Ti6Al4V are good for it when used as hard tissue biomaterial. For example, a textured surface is able to stabilize the bone-implant interface (bonding strength), minimize micro-motion during arthrodesis, promote osseo-integration, carry drug or biomaterial particles, improve antibacterial properties, and enhance the related bio-properties of bioactive coating/film/layer, and so on [69,77,125,126,127,128]. Meanwhile a surface distributed with ordered TiO_2_ nanotubes (NTs) can also be considered as a kind of surface texture. The nanostructure topography, TiO_2_ NTs diameter and length, the spacing in between the nanotubes and the protein physical properties as electric charge and size are all crucial to the interactions of cells, proteins, molecules, and bacteria with textured titanium [129].

In Wang et al.’s work [69] a subsequent treatment of fine particle shot-peening (FPSP) process was performed on micro arc oxidation (MAO) coatings on Ti6Al4V, the obtained rougher dimple surfaces interspersed by fine pore structure was beneficial to induce the deposition of biomimetic apatite. Kumari et al. [77] found that there was an increased bioactivity on LST treated Ti6Al4V surface according to the calcium phosphate deposition rate in Hank’s solution. As expected, LST did not produce any cytotoxic substances reflected by XTT (C_22_H_19_N_7_Na_2_O_14_S_2_) assay test. It was shown that cell adherence preferred ridges and corners and was less in the dimple textured surface, in respect of linear textured surface, cells were preferentially attached along the direction of texturing in the textured zone. Olivares-Navarrete et al. [125] prepared macro/micro/nano-textures on Ti6Al4V surfaces by employment of sand blasting and acid etching. It was seen that average surface roughness played an important role in determining the response between cells in the osteoblast lineage and Ti6Al4V implants. Additionally, macroscale textured surfaces were favorable for mechanical stability during arthrodesis, however, these features generated no positive effect on the healing response at a cellular level. Surface features formed with micro-texture and nano-texture were able to be discriminated by committed osteoblasts and multipotent MSCs, which is to say they were sensitive to surfaces with microscale and nanoscale features. Meng et al. [126] first fabricated blind micro-hole array on Ti6Al4V via LST by trial and error, the formed textured surface obtained from optimal laser parameters was successively coated with hydroxyapatite (HA) coating through electrophoretic deposition technology. It was found that the textured surface was convenient for trapping nano HA particles and depositing a coating on Ti6Al4V. LST + electrophoretic deposition HA coating was considered to improve the carrying capacity of Ti6Al4V used as biomaterial. Inspired by dragonfly wings, Bhadra et al. [127] prepared nano-patterned surface arrays on titanium samples via a facile one-step hydrothermal etching process. The fabricated titanium surfaces revealed similar surface architecture to dragonfly wings, and the received surfaces possessed selective bactericidal activity indicating by reduction of almost 50% of *Pseudomonas aeruginosa* cells and about 20% of the *Staphylococcus aureus* cells, respectively. Kurella et al. [128] conducted laser processing to coat zirconia (ZrO_2_) and texture simultaneously on Ti6Al4V to produce surfaces that were hierarchically integrated and organized at multiple scales. Cataphracted surfaces with high specific surface area were finally obtained on the ZrO_2_ coating. Such chemical and physical transformations were expected in a Ti6Al4V bio-implant, which was good for effective contact with protein, cells, and tissues at various scales and was also helpful to enhance its chemical and mechanical (tribological) performance in the bio-environment.

By Chen et al. [130], the effects of ST on the interactions between human osteosarcoma (HOS) cells and different Ti6Al4V coupons were studied. The Ti6Al4V coupons differed in surface topography: polished Ti6Al4V (control); roughened Ti6Al4V (Al_2_O_3_ blasted), and LST grooved Ti6Al4V samples with controlled interval spacing (20, 30, 40, 50, and 60 μm). Immuno-fluorescence staining of adhesion proteins (actin and vinculin) was applied to investigate the spreading and adhesion of HOS cells in 48-h culture experiments. Quantitative measures of adhesion were also realized by employment of an enzymatic detachment assay. The results revealed that the HOS cells were strongly affected by variations in the ST at micron-scale. Cell spreading on polished and roughened surfaces presented irregular orientations. It was also found that cell spreading reduced with increased surface roughness. After a 2-day culture duration, the stress fibers of the actin cytoskeleton were observed co-localizing with the focal adhesions at the ends of the stress fibers on all the investigated surfaces. On the micro-grooved textured surfaces, actin microfilament alignment reflected the orientation as a whole and focal adhesion concentration was found to scale with the level of contact guidance. Enhanced orientation and attachment were observed on the Ti6Al4V micro-grooved textured surfaces with groove interval spacing of 20 μm and with a micro-roughness characterized by higher rms surface roughness. Contact guidance was revealed to increase as grooved spacing decreased. The lower enzymatic detachment rates obtained for the LST treated Ti6Al4V showed that LST provided improved adhesion between HOS cells and laser textured surfaces. It was also found that ST had a strong effect on cell detachment rates. For the range of micro-grooved geometries studied, micro-grooves with depth of ~10 μm, width of ~11 μm, and interval spacing of 20 μm indicated the most promising combination of cell orientation and adhesion of HOS cells to LST grooved Ti6Al4V surface. Furthermore, Chen et al. [131] conducted the initial cell spreading and adhesion on longitudinally- and transversally-oriented micro-grooved Ti6Al4V surfaces formed by LST. The results showed that cell-spreading and adhesion were both enhanced by longitudinally-oriented and transversally-oriented micro-grooves. Contact guidance was found to promote cell adhesion due to the increasing interactions between the focal adhesions and the patterned extra-cellular matrix (ECM) proteins on the micro-grooved surfaces.

In Fasasi et al.’s work [132] the diode pumped solid-state (DPSS) 355 nm (UV) laser operating with a pulse repetition frequency (PRF) of 50 kHz, a focal length of 100 nm, and scan speeds of ~200 mm/s to 300 mm/s produced micro-groove geometries that were close to the ‘optimal’ groove depth and width of 8 to 12 μm. Such groove dimensions were in the range that could promote cell integration and contact guidance. The results from this study suggest that nano-second DPSS UV lasers can be used to introduce the desired micro-groove geometries without micro-cracks in the heat-affected zones. The desired 8~12 μm groove depths and widths can be achieved by control of pulse frequency, scan speed, and lens focal length that controls spot size. The appearance of the physical surface features (resolidification packets, ripples, and wall deformations) obtained using DPSS UV lasers, warrants further studies. This may lead to further optimized groove geometries that promote increased cell adhesion.

Wettability on the implant material surface which could modulate the protein adsorption and thereby affect cell attachment and tissue integration at the interface, usually plays an important role in the success of an implanting operation. In order to improve the wettability, both the surface topography and the surface chemistry, Dahotre and Paital et al. [133,134] first sprayed Ca-P (calcium phosphate tribasic, Ca_5_(OH)(PO_4_)_3_) slurry onto Ti6Al4V substrate, and then conducted direct laser writing on the Ca-P coating. Various phases such as, CaTiO_3_, Ca_3_(PO_4_)_2_, TiO_2_ (Anatase and Rutile) were detected in the coated regions. The received STs obtained using direct laser writing technique suggested a remarkable decrease in the apparent contact angle to simulated body fluid (SBF) and distilled water. Meanwhile the textured Ca-P coating surface was favorable to cell spreading, which was confirmed by comparative investigations with a representative Ca-P-coated Ti6Al4V in the spreading of the MC3T3-E1 osteoblast cells after culture for 24 h. The behavior of the cells on a surface is significantly influenced by the amount and direction of stress on the cytoskeleton, while the surface chemistry and surface topography also play key roles on cell behavior. Mukherjee et al. [135] found that the surface with narrower or sharper secondary texture features on a groove textured Ti6Al4V surface was able to promote cell attachment and differentiation. As the cell cytoskeleton held low stiffness, getting attached to a surface with sharp features was able to induce the type of stress that was beneficial for the cell activities. A surface with such feature dimensions could be effective in influencing the cellular activities on the surface and thus enhancing the biocompatibility of groove textured Ti6Al4V on the surface. Mirhosseini et al. [136] found that small holes formed by LST on Ti6Al4V samples could not only change the surface roughness in comparison to the shot blasting treated Ti6Al4V, but also created better cell integration and increased 2T3 osteoblast cell growth. LST increased the surface energy of Ti6Al4V and resulted in a more active surface to attach cells.

Anodizing titanium to fabrication nanotubes (NTs) is a simple strategy for providing inexpensive and well-ordered nanotopographies on implant surfaces to enhance their biological behavior. It has been suggested that using anodizing to create NTs on Ti surfaces presents great promise in vitro; it is possible to improve stem cell differentiation, increase bone growth, improve bladder stent urothelialization, increase vascular stent endothelialization, decrease bacteria function and inflammation. Meanwhile the Ti surfaces distributed with NTs produced by anodizing, exhibited continued promise in various orthopedic applications after in vivo estimations [137]. Kummer et al. [138] conducted anodizing on Ti to form TiO_2_ NTs with 20, and 80 nm tube diameters under applied voltages changed to 5, and 20 V, respectively, in a 0.5% hydrofluoric acid electrolyte solution for 30, and 15 min, respectively. The anodizing treated Ti samples were also subjected to heat treatment to remove fluorin. It was revealed that after ultraviolet (UV) light, ethanol soaking, and autoclaving, the treated Ti samples which possessed 20 nm NTs presented the greatest promise as an antibacterial implant material.

Fibronectin and vitronectin are two major proteins which play important roles in osteoblast adhesion. It was reported that TiO_2_ NTs significantly increased fibronectin (15%) and vitronectin (18%) adsorption on anodized titanium in comparison to the raw titanium samples, due to promoting adherence of cells. The fibronectin and vitronectin adsorption step was increased on anodized titanium substrates with TiO_2_ NTs, which benefited from biomechanical interlocking (high specific surface area) and biological interactions (enhance bone cell function) [139].

Balasundaram et al. [140] found that TiO_2_ NTs obtained by anodizing titanium could promote osteoblast adhesion through BMP-2 knuckle peptide functionalization. The received TiO_2_ NTs could also act as drug molecules or bone building agents (such as RGD, KRSR, etc.) carrier for new bone formation. Furtherly, Zile et al. [141] performed functionalization of TiO_2_ NTs with fibroblast growth factor-2 (FGF-2). It was seen that the FGF-2 functionalized TiO_2_ NTs were able to increase keratinocyte density, reduce bacteria adhesion and promote bone tissue formation, as expected.

Liu et al. [142] conducted temperature-controlled atomic layer deposition (ALD) to fabricate unique nano-TiO_2_ coatings on Ti substrates. Increased surface nano-roughness and surface energy contributed to these antibacterial properties. The prepared nano-TiO_2_ coatings showed promising antimicrobial effects against gram-positive bacteria (*S. aureus*), gram-negative bacteria (*E. coli*), and antibiotic-resistant bacteria (MRSA) all without resorting to the application of antibiotics. Meanwhile in vitro results revealed that TiO_2_ coating stimulated osteoblast adhesion and proliferation while suppressing fibroblast adhesion and proliferation, as compared with the original materials.

Bhardwaj et al. [143] obtained a nanophase titanium dioxide surface texture on Ti6Al4V alloy using electrophoretic deposition (EPD). Two distinct nanotopographies (Ti-160 and Ti-120) both presented a certain reduction in *Staphylococcus aureus*, *Pseudomonas aeruginosa,* and *Escherichia coli* compared to the untreated controls. There were 95.6%, 90.2%, and 81.1% reductions for Ti-160 samples, respectively. Similarly, Ti-120 sample respectively displayed reductions of 86.8%, 82.1%, and 48.6%. In addition, osteoblast proliferation on Ti-120 at day 3 and day 5 was increased by 120.7% and 168.7% over the controls.

Hosseini et al. [144] prepared four different photoanodes by sol-gel spin coating onto a glassy substrate of fluorine-doped tin oxide. The photocatalytic activities of TiO_2_, TiO_2_/C/TiO_2_, TiO_2_/C/C/TiO_2_, and TiO_2_/C/TiO_2_/C/TiO_2_ photoanodes were evaluated under UV-Vis light irradiation. A higher photocurrent density was detected with double layers of mesoporous carbon between TiO_2_ as compared with a single layer of mesoporous carbon. A double layer of mesoporous carbon between TiO_2_ also indicated a higher degree of surface roughness in comparison to a single layer of mesoporous carbon. A remarkable improvement in photocurrent was observed by adding additional layers as shown for the two photoanodes: TiO_2_/C/TiO_2_ and TiO_2_/C/TiO_2_/C/TiO_2_. The addition of two carbon layers enhanced the graphite sheets between the TiO_2_ layers. The graphite sheets might facilitate rapid transport of charges or contribute to generation of charged carriers owing to the functional groups of mesoporous carbon.

Hanson et al. [145] used modified poly(L-lactic acid) (PLLA) scaffolds using oxygen plasma treatment to increase surface hydroxyl groups and thereby improve substrate hydrophilicity. The contact angle of water on PLLA decreased from 75.6° to 58.2° after oxygen plasma treatment, which meant the surface hydrophilicity of PLLA was increased. The DNA analysis results suggested that there was an increased number of human mesenchymal stem cells (hMSCs) on oxygen plasma treated scaffolds. Oxygen plasma treatment also promoted a more even distribution of hMSCs throughout the scaffold and enhanced cell spreading at earlier time points without affecting cell viability.

TiO_2_-based nanotubes with high specific surface area and ion-changeable ability have been considered for extensive biomedical applications (osseointegration, antibacterial activity, and drug delivery) [146,147]. Sterilization is usually the final surface treatment procedure of all implantable devices and it also must be considered before implementation. Different sterilization procedures for all implantable devices can influence mechanical properties and biological responses [146,147,148]. Junkar et al. [148] investigated the effect of different sterilization techniques (sterilization with autoclave, sterilization with ultra-violet (UV) light radiations, commercial hydrogen peroxide (H_2_O_2_) plasma, and oxygen plasma treatment) on titanium dioxide nanotubes (TiO_2_ NTs). TiO_2_ NTs with three different diameters of 15 nm (NT15), 50 nm (NT50), and 100 nm (NT100) were prepared using anodization. It was seen that different sterilization procedures did not influence the wettability of TiO_2_ NTs. However, steam autoclaving destroyed the nanostructure of TiO_2_ NTs, while UV-light, commercial H_2_O_2_ plasma sterilization, and oxygen plasma treatment techniques showed no nagetive effects on TiO_2_ NTs surface features.

Kulkarni et al. [149] conducted investigation of protein interactions with layers of TiO_2_ nanotubes (NTs) and nanopores (NPs). The proteins presence on the nanostructures was evaluated by XPS and ToF-SIMS. It was found that there was significant difference in surface charge density between the inner NTs/NPs. NTs adsorbed 31% more histone and albumin than those of NPs. The differences were due to the distribution of NTs: the albumin/histone could also bind to the inner and partially to the outer surface of NTs due to steric and charge restrictions, as compared with the one edge NPs. The size, net charge, and internal charge distribution of proteins had obviously affected their binding ability to the negatively charged TiO_2_ surface. Meanwhile longer NTs which had a higher total surface area, absorbed more protein. NTs with small diameter were able to bind more small-sized positively charged proteins per surface area, e.g., histone. According to theoretical modelling, small diameter TiO_2_ NTs could lead to an increased magnitude of the surface charge density (negative) at the wall edge, which was favorable to more histone adhesion. In addition, protein adhesion on the top surface revealed a higher protein amount on the NTs tops for histone in comparison to albumin. All in all, the importance of TiO_2_ nanostructures’ topography was suggested for biomedical applications such as drug delivery or implant materials, where interactions with small size proteins or molecules were primordial.

Interactions between the implant surface and the surrounding bone tissue are essential for the successful integration of a bone implant. In respect of titanium (Ti) implant, it has been confirmed that the contact between the cell membrane of osteoblasts and the Ti oxide surface is established in two steps: first, the osteoblast’s cell membrane might set a non-specific contact due to electrostatics (originating in the Coulomb interaction between the negatively charged surface and positively charge proteins), followed by a second step, where the specific binding was made [150]. Kabaso et al. [150] found that adhesion of osteoblast-like cells to a Ti surface implant was a dynamic process driven by interaction with the extracellular matrix and intracellular mechanisms after the Monte Carlo (MC) simulations. The free energy of the system was decreased as the osteoblasts bound to the Ti surface. On the other hand, the strong interactions at contact regions could capture the membrane and increased the local lateral membrane tension leading to an increase in free energy of the cell membrane. It was shown that membrane-bound protein complexes (PCs) increased the membrane protrusion growth between the osteoblast and the groove-textured on the titanium (Ti) surface and thereby promoted the adhesion of osteoblasts to the Ti surface.

Hamlekhan et al. [151] optimized the anodizing and annealing conditions to achieve non-aging hydrophilic surfaces (TiO_2_ nanotubular surface) on Ti6Al4V alloy. It was found that the nanotubes obtained by anodizing at 60 V and followed by annealing at 600 ℃ maintained their hydrophilicity significantly longer. Due to presence of nanotubes with larger dimensions and higher surface roughness, 60 V anodized samples revealed a lower water contact angle (WCA). On the other hand, the annealing temperature was the main factor that could affect the maintenance and stability of the obtained hydrophilic TiO_2_ nanotubular surfaces. Anodizing at high voltages partially promoted the formation of a crystalline structure, and then enhanced surface hydrophilicity. It was suggested that as the surface hydroxylation/dehydroxylation equilibrium was reached, the aged surfaces lost their hydrophilicity. Transformation of the amorphous structure to an anatase crystalline structure was able to slow down the hydroxylation/dehydroxylation equilibrium progress on the TiO_2_ nanotubular surface while the slowest equilibrium process occurred as the anatase was transformed to rutile.

Cunha et al. [152] formed textured surfaces on Ti6Al4V by a femtosecond laser treatment. Four types of STs were obtained on Ti6Al4V samples: (ST-1) nanoscale laser-induced periodic surface structures (LIPSS); (ST-2) nanopillars; (ST-3) a bimodal roughness distribution texture formed of LIPSS overlapping microcolumns; (ST-4) a complex texture formed of LIPSS overlapping microcolumns with a periodic variation of the columns size in the laser scanning direction. The roughness values of the textured surfaces were characterized by the arithmetic mean surface roughness (Ra) and the mean peak to valley height (Rz), calculated from the surface profiles. Distilled-deionized (DD) water and Hank’s balanced salt solution (HBSS) were used to evaluate surface wettability of polished and surface textured Ti6Al4V by the sessile drop method. It was found that the surface roughness values of the four STs were as follows: ST-1 Ra = 290 ± 20 nm and Rz = 2.4 ± 0.3 μm; ST-2 Ra = 260 ± 10 nm and Rz = 2.1 ± 0.1 μm; ST-3 Ra = 1.1 ± 0.1 μm and Rz = 8.5 ± 1.0 μm; ST-4 Ra = 4.7 ± 0.6 μm and Rz = 26.8 ± 2.3 μm, respectively. The polished Ti6Al4V was wetted by both liquids and the contact angles were very similar for the two liquids (68.0° and 63.4° for DD water and HBSS, respectively after 600 s of contact). However, the surface textured surfaces revealed a time-dependent wetting behavior. At t = 0 s, besides ST-3, all the surfaces were wetted by water (θ <90°). HBSS could wet all the textured surfaces. ST-1 and ST-4 showed the best wetting for both liquids, with equilibrium contact angles of 43.4° and 24.1° for water and 21.9° and 8.4° for HBSS, respectively. The reduction of the contact angle with HBSS was maximum for surfaces with ST-4 and then reached a very low contact angle of about 8.0° the droplets spread over all the textured surface. ST-2 and ST-3 suggested similar wetting behavior to the control polished one, with equilibrium contact angles of 56.2° and 76.2° for water and 61.8° and 47.6° for HBSS, respectively. It could be concluded that the HBSS spread much faster than water on the textured surfaces, reflecting an average value of the spreading coefficient 60% higher than that of water. The anisotropy of the surfaces was a key factor in controlling the wetting behavior. Surface texturing on Ti6Al4V by femtosecond laser certainly was an effective route to refine its surface wettability, and also held potential application in improving mesenchymal stem cells adhesion when Ti6Al4V was used as biomaterial.

Super-hydrophobicity (water contact angle ≥150° and sliding angle ≤10°) on the surface of Ti6Al4V can also be achieved by surface texture-based surface treatments to meet the required service behaviors in some fields outside of biomaterials [80,81,153,154,155,156,157,158,159,160]. Shen et al. [80] obtained hierarchical surfaces on Ti6Al4V with super-hydrophobicity via a multi-step process: Step-1 traditional sand blasting of the polished Ti6Al4V samples was carried out with aluminum oxide (60 mesh, 150 mesh, and 300 mesh) at 0.5 MPa for 10 s to form uneven rough structured surfaces like microhills (textured surfaces); Step-2 hydrothermal treatment of the sand blasted samples was conducted in an autoclave with 30 mL of 1 M NaOH solution in a 220 ℃ oven and reacted for different times (1 h, 2 h, 4 h, 6 h, 8 h, and 12 h) and cooled down to ambient temperature in the oven. Thereafter the samples were immersed in 1 M HCl solution for 30 min. The samples were rinsed with deionized water and put into a muffle furnace (heating rate was 2 ℃ s^−1^) and heated at 500 ℃ for 3 h leading to the growth of one-dimensional (1D) TiO_2_ nanowires on the surfaces with microscale rough structures. This process significantly increased the specific surface area of the samples, resulting in an increase of grafted area with the fluorine-containing low-surface-energy groups. Step-3 all of the hydrothermally treated samples were immersed in 1 wt % FAS-17 ethanol solution for 24 h and then dried in a 120 ℃ oven for 2 h to obtain the final samples.

Sand blasting with 60 mesh aluminum oxide produced a large-size concave-convex structure (~60 μm). While sand blasting with 150 mesh aluminum oxide received a relatively even concave-convex structure (~35 μm). Sand blasting by employment of 300 mesh aluminum oxide resulted in an even finer structure with a smooth overall topography. As fluorination modifications were conducted on the sand blasted surfaces with FAS-17, hydrophobic surfaces were obtained in comparison to fluorination modification on the polished surface. The sand blasted sample with 150 mesh aluminum oxide followed by fluorination modification showed the largest contact angle of liquid droplets (~135°) among the measured surfaces. The reaction duration of hydrothermal treatment had certain effects on the sizes of the formed nanowires, and furthermore influenced the contact angles of the fluorination modified sand blasted surfaces with 150 mesh aluminum oxide. It was found that with a short hydrothermal reaction time (1–2 h), the formed nanowires were relatively short and small, which led to the surface wetting state in the transition state between the Wenzel wetting state and the Cassie wetting state; the apparent contact angle of the droplets on the surfaces slightly increased from about 135° to 138° (sliding angle 8° to 7 °). As the hydrothermal reaction time was prolonged to 4 h, the length of nanowire increased with relatively even distribution, and the endings gradually gathered, the spacing distance between the nanowires with each other was far less than 100 nm, forming a larger continuous air layer, the wetting regime of the liquid droplets on the surface successfully changed from the Wenzel wetting state to the Cassie wetting sate (apparent contact angle 155° and sliding angle 6°). When the hydrothermal reaction time was kept to 8 h, the nanowire length was further extended, and a more dense distribution could be found on the surfaces of the microscale structures. The generated secondary nanowires and microscale structure could capture a large quantity of air owing to the higher specific surface area, hence the apparent contact angle of droplets on the surfaces presented a noticeable increasing to 161° (sliding angle 3°). It was also found that there was no obvious change in the apparent contact angle and sliding angle as the hydrothermal reaction time was even longer.

In addition, Shen et al. [153] conducted anodic oxidation on sand blasted Ti6Al4V, followed by fluorination modification with FAS-17. The ordered nanotube arrays were formed on sand blasted Ti6Al4V after anodic oxidation, and the formed surface could be considered as some kind of textured surface. As the ordered nanotube arrays were modified by FAS-17, the final surface of Ti6Al4V revealed an apparent contact angle of about 151° and a sliding angle of about 8°. It was seen that sand blasting + anodic oxidation + fluorination modification with FAS-17 was able to endow Ti6Al4V with a certain super-hydrophobicity on the surface.

On the basis of the above results, Shen et al. [80] expanded the research of super-hydrophobicity to icephobicity (anti-icing property) on the surface of Ti6Al4V. The multi-step treatment of sand blasting (with 150 grit alumina at 0.5 MPa for 10 s) + hydrothermal treatment (in 30 mL 1 M NaOH aqueous solution at 220 ℃ for 8 h) + fluorination modification with FAS-17 (in 1 wt % FAS-17 ethanol solution for 24 h and dried at 120 ℃ for 2 h) was conducted on Ti6Al4V. Icephobicity was evaluated by comparing how long the water droplets were maintained before completely freezing on these surfaces under different temperatures (−10 ℃, −20 ℃, and −30 ℃). A self-made ice adhesion strength measurement device including a cooling plate with a temperature in the range from 0 ℃ to −40 ℃ was applied to measure the ice adhesion strength. There were four types of samples tested: the polished Ti6Al4V (S1), the sand blasted+ fluorination modification with FAS-17 treated Ti6Al4V (S2), the hydrothermal treatment + fluorination modification with FAS-17 treated Ti6Al4V (S3), and the entire multi-step treated Ti6Al4V (S4). The results showed that when the icing process of water droplets was performed at −10 ℃, water droplets were quickly frozen on S1 and S2 (11.3 s and 12.1 s), while water droplets took a much longer time to completely freeze on the S3 (623.9 s) and S4 (750.4 s). The ice adhesion strength values of the tested samples could be arranged as follows: S1 (~730 kPa) > S2 (~320 kPa) > S3 (~160 kPa) > S4 (~80 kPa). As water droplets were conducted at −20 ℃ and −30 ℃, the icing-delay durations of water droplets on all surfaces significantly decreased. However, the icing-delay performance of S4 is obviously superior to the S1, S2, and S3 as expected. Meanwhile it was seen that the ice adhesion strength values of the tested samples under −20 ℃ and −30 ℃ slightly increased compared with those at −10 ℃, but the order was not changed. The S4 sample obtained from multi-step treatment of sand blasting + hydrothermal treatment + fluorination modification with FAS-17 suggested the most promising icephobicity.

Additionally, Shen et al. [154] first formed a microscale array textured surface on Ti6Al4V via chemical micromachining, then the textured surface was successively treated by hydrothermal treatment and fluorination modification with FAS-17 using similar processing parameters to the previous research. There were three types of samples tested: the polished + fluorination modification with FAS-17 treat Ti6Al4V (S1), the hydrothermal treatment + fluorination modification with FAS-17 treated Ti6Al4V (S2), and the surface texturing + hydrothermal treatment + fluorination modification with FAS-17 treated Ti6Al4V (S3). It was found that when the icing process of water droplets was measured at −10 ℃, S1 presented a icing-delay time of 13.2 s, which meant the water droplet was quickly frozen on S1, while water droplets took a far longer time to completely freeze on the S2 (599 s) and S3 (765 s). The ice adhesion strength values of the tested samples were ranked in the following sequence: S3 (~70 kPa) < S2 (~190 kPa) < S1 (~700 kPa). Combining with the measurement results of icing process and ice adhesion strength, it was possible to draw a conclusion that Ti6Al4V obtained extraordinary anti-icing property on the surface after surface texturing + hydrothermal treatment + fluorination modification with FAS-17.

By employment of LST technique, Lian et al. [155,156] prepared three kinds of ST on Ti6Al4V surfaces: groove, crosshatch (included angle 90°), and space-lattice (in the shape of frustum or cylinder), respectively. All of the LST treated surfaces and the raw Ti6Al4V were hydroxylation modified on the surfaces by ultraviolet (UV) radiation for 1 h. Then the hydroxylated samples were immersed in a solution of 1 mL isooctane 15 μL 1H, 1H, 2H, 2H-Perfluorooctyltrichlorosilane (FOTS) for 12 h. Self-assembled monolayers (SAMs) were obtained on the blank and textured Ti6Al4V samples. In this work the polished Ti6Al4V showed a close contact angle to the textured Ti6Al4V of 50–70°, which meant they were hydrophilic surfaces. On the contrary, all of the SAMs-coated samples presented hydrophobicity. The contact angles of the water droplet on the tested samples could be arranged as follows: SAMs-space-lattice (151.6°) > SAMs-crosshatch (126.1°) > SAMs-groove (124.8°) > SAMs-Ti6Al4V (117°). SAMs-space-lattice revealed obvious super-hydrophobicity, meanwhile it was seen that the measured angles were more aligned with the Cassie model.

Based on the above results, Lian et al. [157] examined the effects of surface film on super-hydrophobic characteristics of Ti6Al4V samples with a dimple surface texture. Four different self-assembled monolayers (SAMs): 1H, 1H, 2H, 2H-Perfluorodecyltrichlorosilane (FDTS), 1H, 1H, 2H, 2H-Perfluorooctyltrichlorosilane (FOTS), Octadecyltrichlorosilane (OTS) and 3-Mercaptopropy-trimethoxysilan (MPS) were selected. The influence of spacing values (50 μm, 60 μm, 70 μm, 80 μm, 90 μm, 100 μm) on the super-hydrophobicity of the SAMs coated-textured Ti6Al4V was also investigated. The results showed that when the spacing of the dimple surface texture was 50 μm, all the SAMs coated-textured Ti6Al4V samples held super-hydrophobicity indicated by the contact angles which were higher than 150°. The contact angles of the tested samples were in the following sequence: FDTS coated-textured Ti6Al4V > FOTS coated-textured Ti6Al4V > OTS coated-textured Ti6Al4V > MPS coated-textured Ti6Al4V. The maximum contact angle of 164.5° was achieved on the surface of FDTS coated-textured Ti6Al4V. The contact angles of the tested samples decreased with increasing dimple spacing. Even so, the contact angles of the FDTS, FOTS, and OTS coated Ti6Al4V with surface texture were still larger than 150° and kept super-hydrophobicity on these surfaces. However a noticeable decrease in contact angle was found on the surfaces of MPS coated-textured Ti6Al4V samples, when MPS were coated on textured Ti6Al4V samples with dimple spacing values of 60 μm, 70 μm, 80 μm, 90 μm, 100 μm; the observed contact angles were in the range of 145–130°. The MPS coated-textured Ti6Al4V samples with higher dimple spacing values showed certain hydrophobicity on these surfaces.

Further taking full advantage of the existing results above, Lian et al. [158,159] turned to the specific application of Ti6Al4V with super-hydrophobicity in the marine environment. At first, dimple surface texture and surface pattern inspired from shell surface were produced on Ti6Al4V by LST. Then both of the textured surfaces were coated with nano SiO_2_ particles. A low surface energy solution was prepared with 0.05 mL 1H, 1H, 2H, 2H-perfluoroalkyltriethoxysilanes (PFO) and 0.1 mL ethanol. Droplets of the mentioned solution were dropped on the nano SiO_2_ coated samples. The final received Ti6Al4V samples with two kinds of surface texture not only demonstrated excellent super-hydrophobicity, but also exhibited promising performance of antifouling of halobios in comparison to the polished Ti6Al4V after a 45 d exposure test in neritic region. According to the results above, it was clear that surface textured Ti6Al4V could realize that the functions of icephobicity and antifouling were mainly premised on promising super-hydrophobicity [160].

Wael Att et al. [161] discovered that both UVA (ultraviolet light treatment, peak wavelength of 365 nm) and UVC (ultraviolet light treatment, peak wavelength of 365 nm) treatment could convert the 4-week-old titanium surfaces from hydrophobic to superhydrophilic. However, the UVC phototreatment surmounted the innate bioactivity of new surfaces, and the aged surface increased its rat bone marrow-derived osteoblastic cell attachment capacity to a level 50% higher than that of the new surfaces. In addition, proliferation, alkaline phosphatase activity, and mineralization of cells were higher on the UVC-treated 4-week-old surfaces compared with the new surfaces.

## 3. Summary and Outlook

Mmaterial scientists and engineers have long devoted themselves to the design and production of new materials with better properties to meet the increasing challenges and demands over a wide range of applications under special, aggressive and harsh service conditions. Fabrication of coating onto the surfaces of existing materials by employment of surface modification technologies can obtain the expected properties and improve their surface performance. Titanium and its alloys have been rapidly developed as the pure metal first became commercially available after the 1950s. Its excellent advantages make Ti6Al4V titanium alloy valued in the titanium alloy family, and it is the most frequently and successfully used titanium alloy. However, the disadvantages of Ti6Al4V cannot be ignored, Ti6Al4V holds shortcomings of low surface hardness, high friction coefficient, and insufficient abrasive wear resistance. Surface modification technologies are attractive and suitable approaches to solve the above problems that occur on the surface of Ti6Al4V. A series of surface modification technologies have been used to improve the tribological performance of Ti6Al4V. In addition, surface texture which is inspired from the non-smooth surfaces in the natural world has been considered as an effective approach to refine the tribological behavior of materials and tools in tribology-related fields. Meanwhile along with the expansion and deepening of research on surface texture, Ti6Al4V has also benefited a great deal from surface texture in biological-related applications.

In this mini review, the surface texture-based surface treatments on Ti6Al4V for tribological (with/without lubricated, cavitation erosion) and biological (where Ti6Al4V was used as biomaterials and for the applications of super-hydrophobicity, icephobicity and antifouling) applications were suggested and consolidated. The following conclusions and prospects were based on published literature.

(1) As Ti6Al4V was treated by single surface texturing, exceptional properties and fascinating functions were simultaneously imparted. Meanwhile the combination of surface texturing and surface treatment could take advantage of the mentioned techniques and realized a “1 + 1 > 2” effect. The surface performance of Ti6Al4V titanium alloy was further tuned by surface texturing-based surface treatments and resulted in more extensive applications as expected.

(2) The positive effects of the surface texture at macro/micro/nano scales on Ti6Al4V in tribological performance also lay in the following aspects: storing solid lubricant and grease to supply continuous lubrication or re-lubrication, improving the elastohydrodynamic effect under liquid lubrication, capturing wear debris, minimizing abrasive wear, and reducing nominal contact area. Meanwhile it was confirmed that surface treatment could provide a surface texture with a strongly helping hand.

(3) Ti6Al4V with different surface textures on the surface was able to achieve a higher specific surface area, which has had a significant effect on the adhesion and proliferation of cell and antibacterial behavior, hydrophilicity or hydrophobicity. Nano-scale texture seems to be more effective compared with the macro- and micro- scale textures. Textured surface covered by coating/film with certain functions has increasingly influenced and enhanced the performance of Ti6Al4V.

(4) The exceptional performance brought by surface texture has been greatly inspired by the non-smooth surfaces of some flora and fauna in nature. There are many species on the world, the specific property of each species with a non-smooth surface has an important significance for bionic research. Such investigations might make a contribution to enrich the studies and applications of surface texture.

(5) There is no one method that appears to work for all conditions. Therefore, the practical application of surface texture-based surface treatment on Ti6Al4V should be conducted case by case rather than having a direct and indiscriminate adoption. Meanwhile the essence and mechanism of physical and chemical reactions between the surface texture and lubricant, cell, bacteria, and liquid drops that occur on the textured surface are worthy of attention.

(6) Despite the surface texture-based surface treatments on Ti6Al4V which have demonstrated significant advances in the tribological and biological applications, the research in this area is still in an early stage. Establishing material systems with different conpositions and multiple functions on e textured surfaces is helpful to accelerate the practical applications of surface texture-based surface treatments. Besides the conventional way of trial and error, computer simulation and big data technologies are also capable of having potential benefits in the design and application of surface texture-based surface treatments.
